# 2,6-Diacetyl­pyridine–resorcinol (1/1)

**DOI:** 10.1107/S1600536812035131

**Published:** 2012-08-11

**Authors:** Quoc-Cuong Ton, Michael Bolte

**Affiliations:** a Institut für Organische Chemie und Chemische Biologie, Goethe-Universität Frankfurt, Max-von-Laue-Strasse 7, 60438 Frankfurt am Main, Germany; b Institut für Anorganische und Analytische Chemie, Goethe-Universität Frankfurt, Max-von-Laue-Strasse 7, 60438 Frankfurt am Main, Germany

## Abstract

The title co-crystal, C_9_H_9_NO_2_·C_6_H_6_O_2_, is composed of one 2,6-diacetyl­pyridine mol­ecule and one resorcinol mol­ecule as the asymmetric unit. In the 2,6-diacetyl­pyridine mol­ecule, the two carbonyl groups are anti­periplanar to the pyridine N atom. In the crystal, the 2,6-diacetyl­pyridine and resorcinol mol­ecules are connected by two O—H⋯O hydrogen bonds, forming planar chains of alternating components running along [120].

## Related literature

For background to 2,6-diacetyl­pyridine and resorcinol, see: Bacon & Lisher (1980 ▶); MacGillivray *et al.* (2000 ▶); Boldog *et al.* (2004 ▶); Matsumoto *et al.* (2006 ▶); Anwar *et al.* (2007 ▶); Friščić & MacGillivray (2009 ▶).
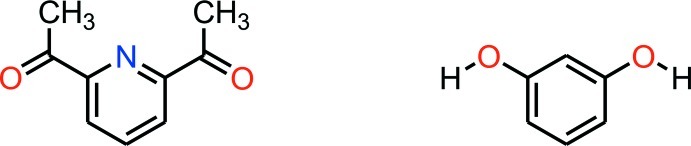


## Experimental

### 

#### Crystal data

C_9_H_9_NO_2_·C_6_H_6_O_2_
*M* *_r_* = 273.28
Triclinic, 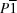

*a* = 7.346 (2) Å
*b* = 7.866 (2) Å
*c* = 12.342 (3) Å
α = 101.61 (3)°
β = 90.51 (3)°
γ = 98.72 (3)°
*V* = 689.9 (3) Å^3^
*Z* = 2
Mo *K*α radiation
μ = 0.10 mm^−1^
*T* = 173 K
0.30 × 0.30 × 0.23 mm

#### Data collection

Stoe IPDS II two-circle diffractometer
9113 measured reflections
2515 independent reflections
1605 reflections with *I* > 2σ(*I*)
*R*_int_ = 0.094

#### Refinement

*R*[*F* ^2^ > 2σ(*F* ^2^)] = 0.050
*wR*(*F* ^2^) = 0.130
*S* = 0.93
2515 reflections
185 parameters
H-atom parameters constrained
Δρ_max_ = 0.19 e Å^−3^
Δρ_min_ = −0.27 e Å^−3^

### 

Data collection: *X-AREA* (Stoe & Cie, 2001 ▶); cell refinement: *X-AREA*; data reduction: *X-RED32* (Stoe & Cie, 2001 ▶); program(s) used to solve structure: *SHELXS97* (Sheldrick, 2008 ▶); program(s) used to refine structure: *SHELXL97* (Sheldrick, 2008 ▶); molecular graphics: *XP* in *SHELXTL-Plus* (Sheldrick, 2008 ▶); software used to prepare material for publication: *publCIF* (Westrip, 2010 ▶).

## Supplementary Material

Crystal structure: contains datablock(s) I, global. DOI: 10.1107/S1600536812035131/ng5287sup1.cif

Structure factors: contains datablock(s) I. DOI: 10.1107/S1600536812035131/ng5287Isup2.hkl

Supplementary material file. DOI: 10.1107/S1600536812035131/ng5287Isup3.cml

Additional supplementary materials:  crystallographic information; 3D view; checkCIF report

## Figures and Tables

**Table 1:** Hydrogen-bond geometry (Å, °)

*D*—H⋯*A*	*D*—H	H⋯*A*	*D*⋯*A*	*D*—H⋯*A*
O3—H*O*3⋯O2	0.84	1.95	2.784 (2)	174
O4—H*O*4⋯O1^i^	0.84	1.96	2.802 (3)	177

Symmetry code: (i) 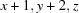.

**Fig. 1.**
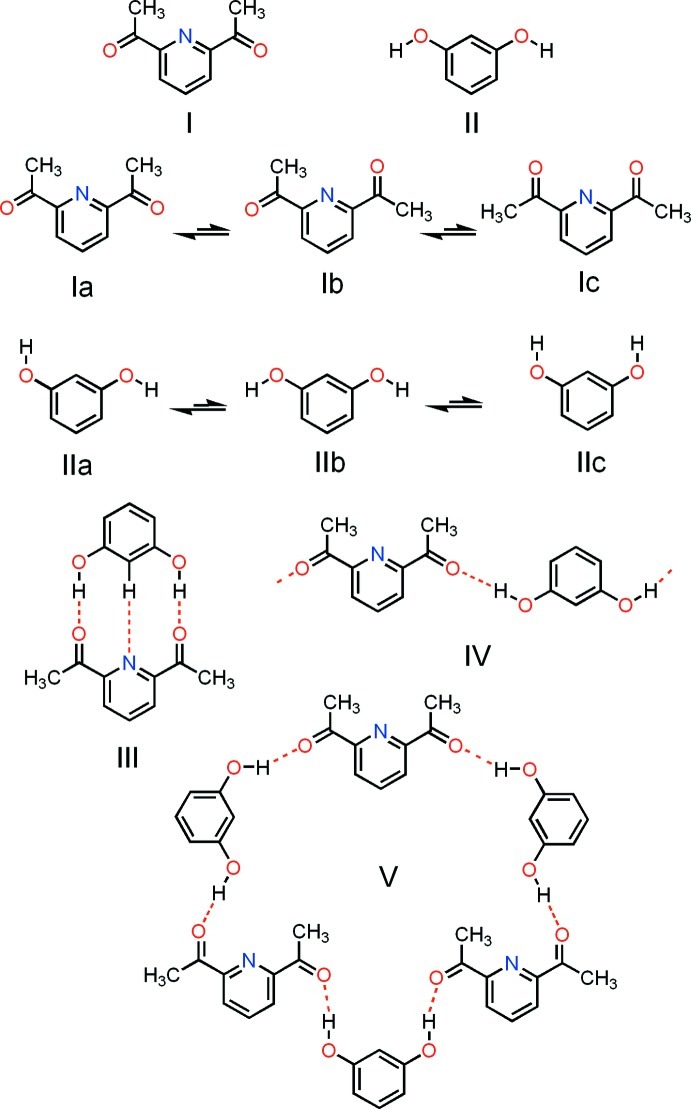
Scheme showing 2,6-diacetylpyridine (I) and resorcinol (II), their possible conformations (Ia, Ib, Ic, IIa, IIb, IIc) and hydrogen bonded complexes of them (III, IV, V).

**Fig. 2.**
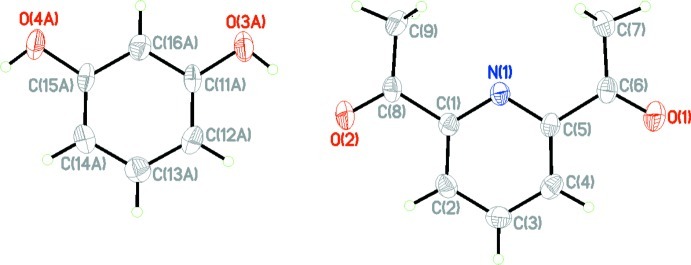
A perspective view of the title complex, showing the atom-numbering scheme. Displacement ellipsoids are drawn at the 50% probability level and H atoms are shown as small spheres of arbitrary radii.

**Fig. 3.**
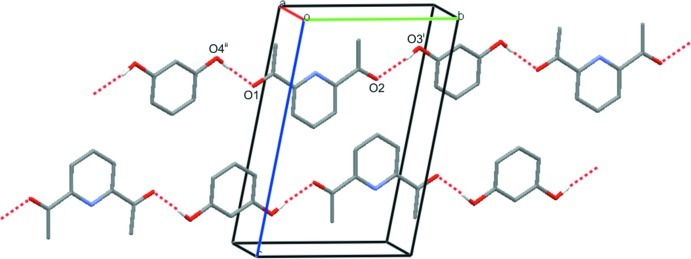
A partial packing diagram of the title complex. Dashed line indicate hydrogen bonds. Only the H atoms involved in hydrogen bonding are shown. [Symmetry codes: (i) -x + 1, -y + 2, -z + 1; (ii) -x, -y, -z + 1.]

## supplementary crystallographic information

### Comment

The co-crystallization process between two components which possess either donor or acceptor hydrogen bond properties in order to obtain the AAA-DDD (A=Acceptor, D= Donor) hydrogen bond pattern containing two strong O—H···O hydrogen bonds and one weak C—H···O hydrogen bond (see Fig. 1, III) was the initial motivation of this research. Therefore, 2,6-diacetylpyridine, I, (CSD REFCODE: BARKAH) and resorcinol, II, (CSD REFCODE: RESORA03) have been chosen for this purpose. Compounds I and II can exist in three possible conformations (Anwar *et al.*, 2007). Considering all possible hydrogen bonds between the two components, forming the complex as mentioned above is the most unfavourable constellation. The calculations of the three molecular conformations of 2,6-diacetylpyridine using quantum-mechanical calculations (Gaussian 03) predict that conformer Ia is the most stable, followed by Ib, and then Ic. The determination of the relative stability of resorcinol using quantum–mechanical density functional theory (DFT) said that conformer IIa is the most stable, followed by IIb, and then IIc. Two out of three conformers of resorcinol have been observed in neutron powder experiments (Bacon & Lisher, 1980). Beyond that all three conformations have been found in diverse multi-component-complexes (Boldog *et al.* 2004; MacGillivray *et al.* 2000; Friščić & MacGillivray, 2009; Matsumoto *et al.*, 2006) where resorcinol showed these conformations. Another possibility of building a finite hydrogen bond network between the two components is highlighted as an example (V in Fig. 1), where different conformers are involved. The energy for the conversion of the relative stable conformers Ia and IIb to the least energetically favoured conformational states Ic and IIc is estimated to be approximately 60 kJ/mol. The co-crystal of the title compound (Fig. 2) in the constellation of Ia and IIb adopts a chain motif (IV in Fig. 1) (Fig. 3). The desired complex (III in Fig. 1) was not formed.

### Experimental

The starting compounds 2,6-diacetylpyridine and resorcinol were purchased from Aldrich and Alfa Aesar which were used for co-crystallization experiments without purification. The starting compounds were dissolved in a 1:1 molecular ratio in ether and setlaid aside at room temperature. After several weeks adequate single crystals were obtained.

### Refinement

All H atoms were refined using a riding model with fixed individual displacements parameters [*U*_iso_(H) = 1.2 *U*_eq_(C) or *U*_iso_(H) = 1.5 *U*_eq_(C_methyl_, O)] with C_aromatic_—H = 0.95 Å, C_methyl_ = 0.98 Å, and O—H = 0.84 Å. The methyl and hydroxyl groups were allowed to rotate but not to tip.

### Crystal data

**Table:**

C_9_H_9_NO_2_·C_6_H_6_O_2_	*Z* = 2
*M**_r_* = 273.28	*F*(000) = 288
Triclinic, *P*1	*D*_x_ = 1.315 Mg m^−^^3^
Hall symbol: -P 1	Mo *K*α radiation, λ = 0.71073 Å
*a* = 7.346 (2) Å	Cell parameters from 9113 reflections
*b* = 7.866 (2) Å	θ = 3.8–25.6°
*c* = 12.342 (3) Å	µ = 0.10 mm^−^^1^
α = 101.61 (3)°	*T* = 173 K
β = 90.51 (3)°	Block, colourless
γ = 98.72 (3)°	0.30 × 0.30 × 0.23 mm
*V* = 689.9 (3) Å^3^	

### Data collection

**Table:**

Stoe IPDS II two-circle diffractometer	1605 reflections with *I* > 2σ(*I*)
Radiation source: fine-focus sealed tube	*R*_int_ = 0.094
Graphite monochromator	θ_max_ = 25.4°, θ_min_ = 3.2°
ω scans	*h* = −8→8
9113 measured reflections	*k* = −9→9
2515 independent reflections	*l* = −14→14

### Refinement

**Table:**

Refinement on *F*^2^	Primary atom site location: structure-invariant direct methods
Least-squares matrix: full	Secondary atom site location: difference Fourier map
*R*[*F*^2^ > 2σ(*F*^2^)] = 0.050	Hydrogen site location: inferred from neighbouring sites
*wR*(*F*^2^) = 0.130	H-atom parameters constrained
*S* = 0.93	*w* = 1/[σ^2^(*F*_o_^2^) + (0.0671*P*)^2^] where *P* = (*F*_o_^2^ + 2*F*_c_^2^)/3
2515 reflections	(Δ/σ)_max_ < 0.001
185 parameters	Δρ_max_ = 0.19 e Å^−^^3^
0 restraints	Δρ_min_ = −0.27 e Å^−^^3^

### Special details

**Table:**

Geometry. All e.s.d.'s (except the e.s.d. in the dihedral angle between two l.s. planes) are estimated using the full covariance matrix. The cell e.s.d.'s are taken into account individually in the estimation of e.s.d.'s in distances, angles and torsion angles; correlations between e.s.d.'s in cell parameters are only used when they are defined by crystal symmetry. An approximate (isotropic) treatment of cell e.s.d.'s is used for estimating e.s.d.'s involving l.s. planes.
Refinement. Refinement of *F*^2^ against ALL reflections. The weighted *R*-factor *wR* and goodness of fit *S* are based on *F*^2^, conventional *R*-factors *R* are based on *F*, with *F* set to zero for negative *F*^2^. The threshold expression of *F*^2^ > σ(*F*^2^) is used only for calculating *R*-factors(gt) *etc*. and is not relevant to the choice of reflections for refinement. *R*-factors based on *F*^2^ are statistically about twice as large as those based on *F*, and *R*- factors based on ALL data will be even larger.

### Fractional atomic coordinates and isotropic or equivalent isotropic displacement parameters (Å^2^)

**Table:**

	*x*	*y*	*z*	*U*_iso_*/*U*_eq_	
N1	0.2051 (2)	0.1792 (2)	0.23716 (14)	0.0241 (4)	
O1	0.0057 (2)	−0.2404 (2)	0.27810 (15)	0.0442 (5)	
O2	0.4266 (2)	0.6213 (2)	0.27795 (14)	0.0421 (5)	
C1	0.2841 (2)	0.3381 (3)	0.29483 (18)	0.0244 (5)	
C2	0.3093 (3)	0.3761 (3)	0.40993 (19)	0.0290 (5)	
H2	0.3624	0.4906	0.4478	0.035*	
C3	0.2559 (3)	0.2451 (3)	0.46820 (19)	0.0323 (5)	
H3	0.2723	0.2674	0.5465	0.039*	
C4	0.1779 (3)	0.0805 (3)	0.40953 (19)	0.0304 (5)	
H4	0.1426	−0.0131	0.4469	0.036*	
C5	0.1518 (2)	0.0537 (3)	0.29475 (18)	0.0243 (5)	
C6	0.0608 (3)	−0.1218 (3)	0.22941 (19)	0.0283 (5)	
C7	0.0371 (3)	−0.1447 (3)	0.1066 (2)	0.0385 (6)	
H7A	0.0046	−0.2700	0.0734	0.058*	
H7B	0.1526	−0.0971	0.0767	0.058*	
H7C	−0.0614	−0.0821	0.0892	0.058*	
C8	0.3460 (3)	0.4780 (3)	0.22922 (19)	0.0277 (5)	
C9	0.3053 (3)	0.4348 (3)	0.1071 (2)	0.0371 (6)	
H9A	0.3542	0.5361	0.0752	0.056*	
H9B	0.1717	0.4063	0.0924	0.056*	
H9C	0.3634	0.3336	0.0735	0.056*	
O3	0.5190 (2)	0.8749 (2)	0.15119 (15)	0.0440 (5)	
HO3	0.4909	0.8049	0.1937	0.066*	
O4	0.8147 (2)	1.4495 (2)	0.14755 (14)	0.0437 (5)	
HO4	0.8697	1.5413	0.1891	0.066*	
C11	0.6121 (3)	1.0305 (3)	0.2107 (2)	0.0292 (5)	
C12	0.6507 (3)	1.0573 (3)	0.3241 (2)	0.0335 (6)	
H12	0.6119	0.9671	0.3635	0.040*	
C13	0.7473 (3)	1.2191 (3)	0.3788 (2)	0.0345 (6)	
H13	0.7743	1.2385	0.4561	0.041*	
C14	0.8046 (3)	1.3522 (3)	0.3220 (2)	0.0331 (6)	
H14	0.8705	1.4616	0.3600	0.040*	
C15	0.7642 (3)	1.3232 (3)	0.2090 (2)	0.0301 (5)	
C16	0.6671 (3)	1.1633 (3)	0.1528 (2)	0.0331 (5)	
H16	0.6386	1.1448	0.0757	0.040*	

### Atomic displacement parameters (Å^2^)

**Table:**

	*U*^11^	*U*^22^	*U*^33^	*U*^12^	*U*^13^	*U*^23^
N1	0.0187 (8)	0.0236 (10)	0.0286 (10)	−0.0016 (7)	0.0019 (7)	0.0060 (8)
O1	0.0515 (10)	0.0298 (9)	0.0484 (11)	−0.0118 (8)	0.0009 (8)	0.0150 (8)
O2	0.0482 (9)	0.0262 (9)	0.0464 (11)	−0.0139 (7)	−0.0057 (8)	0.0094 (8)
C1	0.0179 (9)	0.0252 (11)	0.0290 (13)	−0.0012 (8)	0.0001 (8)	0.0063 (9)
C2	0.0250 (10)	0.0265 (12)	0.0322 (13)	−0.0015 (9)	−0.0039 (9)	0.0028 (10)
C3	0.0317 (11)	0.0377 (13)	0.0260 (13)	0.0021 (10)	−0.0004 (9)	0.0057 (10)
C4	0.0265 (11)	0.0329 (12)	0.0337 (14)	0.0004 (9)	0.0051 (9)	0.0144 (11)
C5	0.0196 (10)	0.0227 (11)	0.0308 (13)	0.0002 (8)	0.0039 (9)	0.0084 (9)
C6	0.0219 (10)	0.0249 (12)	0.0379 (14)	−0.0007 (8)	0.0021 (9)	0.0092 (10)
C7	0.0447 (13)	0.0281 (12)	0.0378 (15)	−0.0061 (10)	−0.0035 (11)	0.0041 (11)
C8	0.0224 (10)	0.0230 (11)	0.0372 (14)	−0.0009 (8)	0.0005 (9)	0.0083 (10)
C9	0.0431 (13)	0.0334 (13)	0.0348 (14)	−0.0040 (10)	0.0023 (11)	0.0147 (11)
O3	0.0492 (10)	0.0281 (9)	0.0507 (11)	−0.0123 (7)	−0.0084 (8)	0.0123 (8)
O4	0.0503 (10)	0.0290 (9)	0.0481 (11)	−0.0114 (8)	0.0098 (8)	0.0121 (8)
C11	0.0219 (10)	0.0217 (11)	0.0431 (15)	−0.0028 (8)	0.0019 (9)	0.0085 (10)
C12	0.0302 (11)	0.0306 (13)	0.0442 (16)	0.0046 (9)	0.0037 (10)	0.0180 (11)
C13	0.0319 (12)	0.0353 (13)	0.0367 (14)	0.0066 (10)	−0.0023 (10)	0.0077 (11)
C14	0.0267 (11)	0.0250 (12)	0.0454 (16)	0.0010 (9)	−0.0005 (10)	0.0043 (11)
C15	0.0230 (10)	0.0234 (11)	0.0446 (15)	−0.0008 (8)	0.0091 (10)	0.0114 (10)
C16	0.0303 (11)	0.0321 (13)	0.0367 (14)	0.0011 (9)	0.0052 (10)	0.0095 (11)

### Geometric parameters (Å, º)

**Table:**

N1—C5	1.343 (2)	C9—H9A	0.9800
N1—C1	1.349 (3)	C9—H9B	0.9800
O1—C6	1.229 (2)	C9—H9C	0.9800
O2—C8	1.226 (3)	O3—C11	1.371 (3)
C1—C2	1.396 (3)	O3—HO3	0.8400
C1—C8	1.512 (3)	O4—C15	1.377 (2)
C2—C3	1.383 (3)	O4—HO4	0.8400
C2—H2	0.9500	C11—C12	1.392 (3)
C3—C4	1.385 (3)	C11—C16	1.393 (3)
C3—H3	0.9500	C12—C13	1.397 (3)
C4—C5	1.397 (3)	C12—H12	0.9500
C4—H4	0.9500	C13—C14	1.390 (3)
C5—C6	1.505 (3)	C13—H13	0.9500
C6—C7	1.496 (3)	C14—C15	1.389 (3)
C7—H7A	0.9800	C14—H14	0.9500
C7—H7B	0.9800	C15—C16	1.393 (3)
C7—H7C	0.9800	C16—H16	0.9500
C8—C9	1.494 (3)		
			
C5—N1—C1	117.35 (18)	C9—C8—C1	118.19 (19)
N1—C1—C2	122.87 (19)	C8—C9—H9A	109.5
N1—C1—C8	117.01 (19)	C8—C9—H9B	109.5
C2—C1—C8	120.12 (19)	H9A—C9—H9B	109.5
C3—C2—C1	119.2 (2)	C8—C9—H9C	109.5
C3—C2—H2	120.4	H9A—C9—H9C	109.5
C1—C2—H2	120.4	H9B—C9—H9C	109.5
C2—C3—C4	118.4 (2)	C11—O3—HO3	109.5
C2—C3—H3	120.8	C15—O4—HO4	109.5
C4—C3—H3	120.8	O3—C11—C12	122.26 (19)
C3—C4—C5	119.18 (19)	O3—C11—C16	117.1 (2)
C3—C4—H4	120.4	C12—C11—C16	120.7 (2)
C5—C4—H4	120.4	C11—C12—C13	118.9 (2)
N1—C5—C4	122.97 (19)	C11—C12—H12	120.6
N1—C5—C6	116.70 (19)	C13—C12—H12	120.6
C4—C5—C6	120.33 (18)	C14—C13—C12	121.1 (2)
O1—C6—C7	122.1 (2)	C14—C13—H13	119.4
O1—C6—C5	119.5 (2)	C12—C13—H13	119.4
C7—C6—C5	118.45 (18)	C15—C14—C13	119.2 (2)
C6—C7—H7A	109.5	C15—C14—H14	120.4
C6—C7—H7B	109.5	C13—C14—H14	120.4
H7A—C7—H7B	109.5	O4—C15—C14	122.4 (2)
C6—C7—H7C	109.5	O4—C15—C16	116.9 (2)
H7A—C7—H7C	109.5	C14—C15—C16	120.7 (2)
H7B—C7—H7C	109.5	C15—C16—C11	119.4 (2)
O2—C8—C9	122.80 (19)	C15—C16—H16	120.3
O2—C8—C1	119.0 (2)	C11—C16—H16	120.3
			
C5—N1—C1—C2	−0.9 (3)	N1—C1—C8—O2	−176.44 (19)
C5—N1—C1—C8	179.22 (17)	C2—C1—C8—O2	3.7 (3)
N1—C1—C2—C3	1.7 (3)	N1—C1—C8—C9	3.7 (3)
C8—C1—C2—C3	−178.40 (19)	C2—C1—C8—C9	−176.1 (2)
C1—C2—C3—C4	−0.4 (3)	O3—C11—C12—C13	179.6 (2)
C2—C3—C4—C5	−1.6 (3)	C16—C11—C12—C13	−0.7 (3)
C1—N1—C5—C4	−1.2 (3)	C11—C12—C13—C14	0.1 (3)
C1—N1—C5—C6	178.93 (17)	C12—C13—C14—C15	0.2 (3)
C3—C4—C5—N1	2.5 (3)	C13—C14—C15—O4	179.3 (2)
C3—C4—C5—C6	−177.67 (19)	C13—C14—C15—C16	0.2 (3)
N1—C5—C6—O1	−178.45 (19)	O4—C15—C16—C11	−179.94 (19)
C4—C5—C6—O1	1.7 (3)	C14—C15—C16—C11	−0.8 (3)
N1—C5—C6—C7	0.3 (3)	O3—C11—C16—C15	−179.2 (2)
C4—C5—C6—C7	−179.55 (19)	C12—C11—C16—C15	1.0 (3)

### Hydrogen-bond geometry (Å, º)

**Table:**

*D*—H···*A*	*D*—H	H···*A*	*D*···*A*	*D*—H···*A*
O3—H*O*3···O2	0.84	1.95	2.784 (2)	174
O4—H*O*4···O1^i^	0.84	1.96	2.802 (3)	177

Symmetry code: (i) *x*+1, *y*+2, *z*.
